# Low-Volume Versus Standard-Volume Pericapsular Nerve Group Block with and Without Dexamethasone for Postoperative Analgesia After Total Hip Arthroplasty: A Randomized Controlled Trial

**DOI:** 10.3390/jcm15103676

**Published:** 2026-05-11

**Authors:** Tomasz Reysner, Bahadir Ciftci, Selcuk Alver, Katarzyna Wieczorowska-Tobis, Małgorzata Reysner

**Affiliations:** 1 Pathophysiology of Pain Unit, Department of Anesthesiology and Intensive Therapy, Poznan University of Medical Sciences, 61-701 Poznan, Poland; treysner@ump.edu.pl; 2 Department of Anatomy, Istanbul Medipol University, 34726 Istanbul, Turkey; 3 Department of Anesthesiology and Reanimation, Istanbul Medipol University, 34214 Istanbul, Turkey; 4 Department of Anesthesiology and Reanimation, Biruni University Hospital, 34295 Istanbul, Turkey; 5 Department of Palliative Medicine, Poznan University of Medical Sciences, 61-701 Poznan, Poland; 6 Department of Clinical Anesthesiology and Pain Management, Poznan University of Medical Sciences, 61-701 Poznan, Poland

**Keywords:** nerve block, hip arthroplasty, dexamethasone, analgesia, ropivacaine, opioid-related disorders

## Abstract

**Background:** The pericapsular nerve group block provides motor-sparing analgesia after total hip arthroplasty, but the optimal injectate volume and the role of dexamethasone remain unclear. **Methods:** In this prospective, randomized, quadruple-blinded trial, 120 patients undergoing total hip arthroplasty under spinal anesthesia were allocated to receive ultrasound-guided pericapsular nerve group block with 20 mL or 10 mL of 0.2% ropivacaine, with or without 4 mg dexamethasone. The primary outcome was time to first rescue opioid within 24 h. Secondary outcomes included opioid consumption, pain scores, quadriceps strength, inflammatory markers, and safety. The study was designed as a superiority trial and was not powered to assess non-inferiority between volume groups. **Results:** Dexamethasone significantly prolonged time to first rescue opioid (hazard ratio 0.08, 95% confidence interval 0.04–0.16; *p* < 0.0001). Injectate volume had no significant effect on the primary outcome (*p* = 0.13), and no interaction between dexamethasone and volume was observed. Total opioid consumption at 48 h was lower in patients receiving dexamethasone (mean difference −3.88 mg oral morphine equivalents; *p* < 0.0001). Pain scores were consistently lower with dexamethasone, while injectate volume had no effect. Quadriceps strength was preserved in all groups. No nerve injury or clinically relevant hyperglycemia was observed. **Conclusions:** Dexamethasone improves analgesic efficacy of the pericapsular nerve group block after total hip arthroplasty. Reducing injectate volume from 20 mL to 10 mL did not significantly affect the analgesic outcomes; however, the study was not designed to assess non-inferiority. These findings support a strategy of volume optimization in regional anesthesia.

## 1. Introduction

Total hip arthroplasty (THA) is among the most commonly performed orthopedic procedures worldwide and is often associated with moderate to severe postoperative pain [1]. Effective analgesia is essential not only for patient comfort but also for early mobilization, functional recovery, and a reduction in perioperative complications [2]. In recent years, there has been a growing emphasis on regional anesthesia techniques that provide effective pain relief while preserving motor function and minimizing opioid requirements [3]. Recent epidemiological data indicate a continuous global increase in the number of total hip arthroplasty procedures, driven by population aging and expanded surgical indications.

The pericapsular nerve group (PENG) block has emerged as a valuable component of multimodal analgesia for hip surgery. By targeting the articular branches innervating the anterior hip capsule, the PENG block provides effective analgesia with minimal impact on quadriceps strength [4]. However, despite its widespread adoption, considerable variability exists regarding the optimal volume of local anesthetic and the role of adjuvants in this block [5].

Higher volumes of local anesthetic are often used in fascial plane blocks to ensure adequate spread; however, this practice may increase the risk of unintended motor blockade or systemic toxicity. Conversely, lower volumes may compromise block duration or analgesic efficacy [6,7]. Therefore, identifying strategies that allow for volume reduction without sacrificing analgesic quality is of significant clinical interest.

Perineural dexamethasone has been increasingly studied as an adjuvant to peripheral nerve blocks [8,9]. Multiple randomized trials and meta-analyses have demonstrated that dexamethasone prolongs block duration and reduces postoperative opioid consumption [10,11,12]. Proposed mechanisms include the modulation of neural inflammation, inhibition of ectopic neuronal discharge, and vasoconstrictive effects that slow local anesthetic absorption [13]. Despite these promising properties, the role of dexamethasone in optimizing PENG block protocols—particularly in the context of volume reduction—remains insufficiently explored.

Moreover, while the analgesic effects of dexamethasone are well-described, concerns persist regarding potential adverse effects, including hyperglycemia and neurotoxicity, especially in elderly patients undergoing major joint surgery [9]. High-quality randomized data addressing both efficacy and safety in this specific clinical context are limited.

The present study was designed to investigate whether the addition of perineural dexamethasone enhances the analgesic efficacy of the PENG block and permits a reduction in local anesthetic volume without compromising pain control, motor function, or safety.

The primary objective was to assess whether perineural dexamethasone prolongs the duration of postoperative analgesia, defined as time to first rescue opioid within 24 h after total hip arthroplasty.

We hypothesized that the addition of perineural dexamethasone would result in a longer time to first rescue opioid compared with the PENG block without dexamethasone.

As a secondary objective, we explored whether reducing the injectate volume from 20 mL to 10 mL would preserve analgesic efficacy when dexamethasone was added.

## 2. Methods

### 2.1. Study Design and Ethical Approval

This study was designed as a prospective, randomized, quadruple-blinded, parallel-group, clinical trial. The trial was prospectively registered at ClinicalTrials.gov (NCT07023120; https://clinicaltrials.gov/study/NCT07023120?term=NCT07023120&rank=1) accessed on 8 June 2025, prior to the first participant’s enrollment (first patient enrolled on 25 June 2025).

The study protocol was approved by the Bioethics Committee at Poznan University of Medical Sciences (approval no. 107/24), chaired by Prof. dr hab. Maciej Krawczyński, on 7 March 2024. Patient enrollment commenced on 25 June 2025. Written informed consent was obtained from all participants before inclusion. The trial was conducted in accordance with the Declaration of Helsinki and Good Clinical Practice guidelines.

### 2.2. Participants

Adult patients scheduled for primary unilateral total hip arthroplasty (THA) under spinal anesthesia were screened for eligibility. Inclusion criteria comprised American Society of Anesthesiologists (ASA) physical status I–III, body mass index between 18 and 35 kg/m^2^, and the ability to understand and use the numerical rating scale (NRS) for pain assessment.

Patients were excluded if they had known hypersensitivity to ropivacaine or dexamethasone, pre-existing neurological deficits affecting the operated limb, chronic opioid use (>30 mg oral morphine equivalents daily for more than one month), coagulopathy precluding regional anesthesia, uncontrolled diabetes mellitus, active infection, pregnancy or breastfeeding, previous surgery on the ipsilateral hip, or inability to comply with postoperative assessments.

### 2.3. Randomization and Blinding

Eligible patients were randomly assigned in a 1:1:1:1 allocation ratio to one of four study groups using a computer-generated randomization sequence. Randomization was performed without stratification and with equal block sizes to ensure balanced group allocation throughout the enrollment period.

Allocation concealment was achieved using sequentially numbered, sealed, opaque envelopes that were opened only after patient enrollment and immediately prior to preparation of the study medication.

The trial employed a quadruple-blind design. Participants, anesthesiologists responsible for intraoperative and postoperative patient management, investigators involved in data collection, and outcome assessors remained blinded to group allocation throughout the study period.

Study solutions were prepared by an independent anesthesiologist who was not involved in patient care, intraoperative management, postoperative assessments, or data analysis. All study medications were prepared in identical syringes with equal appearance and labeling to maintain blinding. Unblinding was permitted only in the event of a serious adverse event requiring knowledge of the administered intervention.

### 2.4. Interventions

All patients underwent standardized spinal anesthesia with 0.5% ropivacaine, administered at a dose of 15–20 mg (3–4 mL), in accordance with institutional practice. After confirmation of an effective sensory and motor spinal block and prior to surgical incision, an ultrasound-guided pericapsular nerve group (PENG) block was performed as part of the multimodal analgesic regimen.

According to group allocation, patients received one of four active comparator interventions. Patients assigned to the PENG 20 mL group received an ultrasound-guided PENG block using 20 mL of 0.2% ropivacaine. Patients in the PENG 20 mL + DEX group received an ultrasound-guided PENG block using 20 mL of 0.2% ropivacaine combined with 4 mg of perineural dexamethasone. In the PENG 10 mL group, the PENG block was performed with 10 mL of 0.2% ropivacaine. Patients allocated to the PENG 10 mL + DEX group received an ultrasound-guided PENG block using 10 mL of 0.2% ropivacaine supplemented with 4 mg of perineural dexamethasone.

In all groups, the study solution was administered as a single-injection PENG block, with the injectate deposited in the pericapsular fascial plane under continuous ultrasound visualization. No additional peripheral nerve blocks were performed in any study group.

### 2.5. Postoperative Analgesia

Postoperative analgesia followed a standardized multimodal protocol applied uniformly across all study groups. All patients received regular non-opioid analgesics, consisting of intravenous paracetamol 1.0 g and intravenous metamizole 1.0 g, administered at fixed, equal time intervals during the postoperative period.

Non-steroidal anti-inflammatory drugs were not administered routinely, taking into account the advanced age of the study population and potential safety concerns related to renal, gastrointestinal, and cardiovascular adverse effects.

Rescue opioid analgesia was provided on patient request or when the numerical rating scale (NRS) pain score exceeded 3. Intravenous morphine was used as the rescue opioid, and all administered opioid doses were converted to oral morphine equivalents for subsequent analysis.

### 2.6. Outcomes

The primary outcome of the study was the time to first rescue opioid administration within the first 24 h after surgery, defined as the interval from completion of the surgical procedure to the first administration of opioid analgesia requested by the patient or required according to the analgesic protocol. The primary analysis was based on a factorial design assessing the main effect of dexamethasone and injectate volume, rather than pairwise comparisons between individual groups.

Secondary outcomes included the total opioid consumption during the first 48 postoperative hours, expressed as oral morphine equivalents. Postoperative pain intensity was assessed using the numerical rating scale (NRS, 0–10) at 4, 8, 12, 24, and 48 h after surgery. Quadriceps muscle strength was evaluated at the same time points using the Medical Research Council (MRC) scale, focusing on knee extension and hip adduction in order to assess motor function preservation.

In addition, systemic inflammatory response was assessed by measuring the neutrophil-to-lymphocyte ratio (NLR) and the platelet-to-lymphocyte ratio (PLR) at 12, 24, and 48 h postoperatively. Blood glucose levels were also recorded at 12, 24, and 48 h after surgery to evaluate the metabolic response. Safety outcomes included the assessment of potential nerve injury using a predefined nerve injury score (N0–N4), evaluated during the first 48 postoperative hours.

### 2.7. Sample Size Calculation

Sample size estimation was based on pilot data obtained at our institution in 20 patients undergoing total hip arthroplasty, with 5 patients included in each study group. In the pilot cohort, time to first rescue opioid administration was consistently shorter in the non-dexamethasone groups (PENG 20 mL: 8.1, 8.8, 9.2, 9.7, and 10.1 h; PENG 10 mL: 7.9, 8.5, 8.9, 9.1, and 9.8 h), with no censored observations, whereas longer times and right-censoring at 24 h were observed in the dexamethasone-containing groups (PENG 20 mL + DEX: 13.5, 14.8, 15.2, 16.0, and 24.0 h; PENG 10 mL + DEX: 13.2, 14.4, 15.0, 15.6, and 24.0 h), where 24.0 h indicated patients remaining opioid-free during the observation period. Differences between the 10 mL and 20 mL injectate-volume protocols were minimal, whereas a clear separation between dexamethasone-containing and non-dexamethasone groups was observed.

The trial was powered for the primary endpoint, defined as time to first rescue opioid administration within 24 h after surgery. Sample size estimation was performed within a time-to-event framework using a log-rank test, based on the expected separation of Kaplan–Meier survival curves between dexamethasone-containing and non-dexamethasone groups, and accounting for right-censoring.

Assuming a two-sided alpha level of 0.05 and 80% statistical power, and considering the magnitude of effect observed in the pilot data (early events in non-dexamethasone groups and delayed or censored events in dexamethasone groups), it was estimated that 30 patients per group would be sufficient to detect a clinically meaningful difference in the primary outcome. Given the four-arm parallel-group design, the total planned sample size was therefore 120 patients. The study was powered for the primary endpoint but not for rare adverse events or small differences in exploratory secondary outcomes.

### 2.8. Statistical Analysis

All analyses were performed according to a prespecified statistical analysis plan. The primary analysis followed a modified intention-to-treat principle and included all randomized patients who received the allocated intervention.

Continuous variables are presented as means with standard deviations or medians with interquartile ranges, as appropriate, and categorical variables as counts with percentages. Distributional assumptions were assessed using the Shapiro–Wilk test and visual inspection of histograms and Q–Q plots.

#### 2.8.1. Primary Outcome

The primary outcome, time to first rescue opioid administration within 24 h, was analyzed as a time-to-event variable. Patients who did not receive a rescue opioid within 24 h were treated as right-censored observations at 24 h.

Time-to-event data were analyzed using Kaplan–Meier survival curves, and differences between groups were assessed using the log-rank test. Hazard ratios (HRs) and corresponding 95% confidence intervals (CIs) were estimated using a Cox proportional hazards model. Because a high proportion of patients in the dexamethasone groups remained opioid-free at 24 h, standard Cox regression was affected by quasi-complete separation. Therefore, a penalized Cox proportional hazards model with ridge penalization was used to obtain stable estimates. The penalized Cox model included dexamethasone use (yes/no), injectate volume (10 mL vs. 20 mL), and their interaction as fixed effects. Penalized Cox regression was applied due to quasi-complete separation arising from a high proportion of censored observations in the dexamethasone groups, which may lead to instability in standard maximum-likelihood estimates. The proportional hazards assumption was assessed and not violated.

#### 2.8.2. Secondary Outcomes

Total 48-h opioid consumption and other continuous outcomes were analyzed using a mixed-effects model with dexamethasone use (yes/no) and injectate volume (10 mL vs. 20 mL) as fixed factors, including their interaction term. Repeated measurements of pain intensity (NRS), quadriceps strength, inflammatory markers (NLR, PLR), and blood glucose levels were analyzed at each time point using two-way ANOVA with the same factorial structure. Categorical outcomes were compared using the chi-square test or Fisher’s exact test, as appropriate. Safety outcomes, including nerve injury, were analyzed descriptively because no events occurred. Given the balanced factorial design and prespecified postoperative assessment times, separate analyses at each time point were considered appropriate for the secondary repeated-measures analyses.

#### 2.8.3. Multiple Comparisons and Statistical Significance

Bonferroni correction was applied to post hoc comparisons of secondary outcomes across multiple time points (NRS, NLR, PLR), while primary outcome analysis was not adjusted. Statistical analyses were performed using (GraphPad Prism version 10.0; GraphPad Software version 11.0.1, San Diego, CA, USA). Results are reported in accordance with CONSORT guidelines, with effect sizes presented alongside 95% confidence intervals where appropriate.

## 3. Results

### 3.1. Participant Flow and Baseline Characteristics

**Figure 1:**
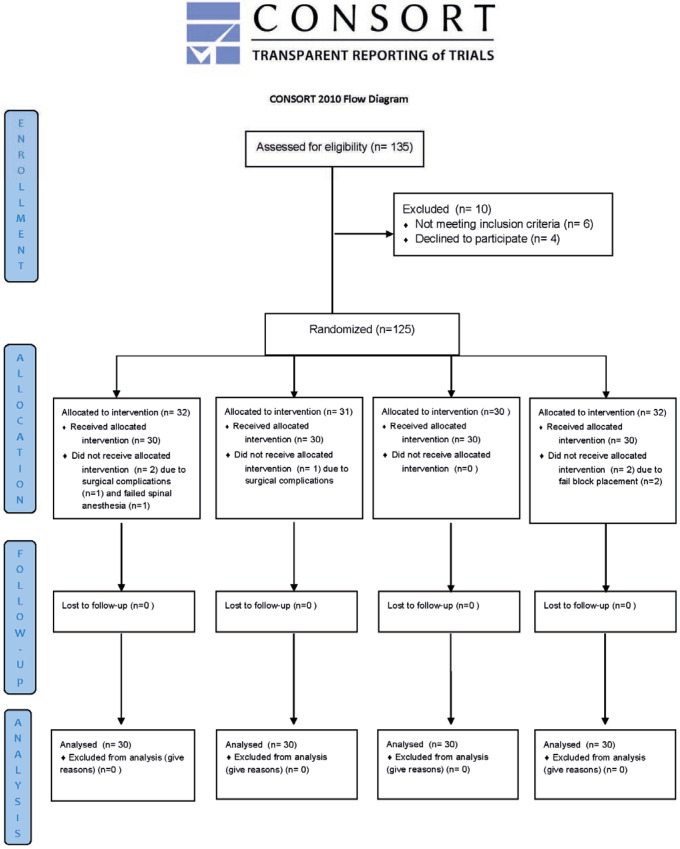
CONSORT flow diagram of patient enrollment, randomization, allocation, follow-up, and analysis.

A total of 135 patients were assessed for eligibility. Ten patients were excluded prior to randomization, including six who did not meet the inclusion criteria and four who declined to participate. Ultimately, 125 patients were randomized into four study groups. A total of 32, 31, 30, and 32 patients were allocated to each group, respectively. After allocation, five patients did not receive the assigned intervention due to intraoperative or anesthetic issues (surgical complications, failed spinal anesthesia, or failed block placement). No patients were lost to follow-up. Consequently, 120 patients (30 per group) completed the study and were included in the final analysis (Figure 1).

Baseline demographic and clinical characteristics were comparable across all four groups. There were no significant differences in age, sex distribution, body mass index, ASA physical status, duration of surgery, or side of surgery, indicating appropriate randomization and group balance (Table 1).

### 3.2. Primary Outcome

**Figure 2:**
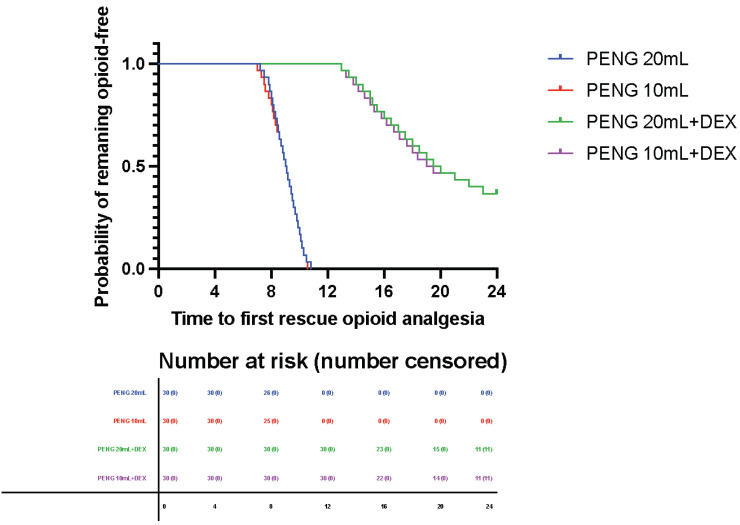
Time to first rescue opioid administration within 24 h.

Time to first rescue opioid administration differed significantly between groups and was primarily driven by the use of perineural dexamethasone. Kaplan–Meier analysis demonstrated clear separation of the survival curves between the dexamethasone-containing and non-dexamethasone groups, indicating a marked prolongation of opioid-free time in patients receiving dexamethasone (log-rank *p* < 0.0001) (Figure 2).

Kaplan–Meier curves showing time to first rescue opioid administration across the four study groups: PENG 20 mL, PENG 20 mL + dexamethasone, PENG 10 mL, and PENG 10 mL + dexamethasone (Table 2).

A substantial proportion of patients in the dexamethasone groups remained opioid-free throughout the 24-h observation period, whereas most patients in the non-dexamethasone groups required rescue opioid within the first postoperative hours. Injectate volume (10 mL vs. 20 mL) did not meaningfully influence time to first rescue opioid, as reflected by the overlapping survival curves within both dexamethasone and non-dexamethasone strata.

In the penalized Cox proportional hazards model, perineural dexamethasone was associated with a markedly reduced hazard of first rescue opioid administration within 24 h (HR 0.08, 95% CI 0.04–0.16; *p* < 0.0001). Injectate volume had no significant effect on the hazard of opioid requirement (*p* = 0.1267), and no significant interaction between dexamethasone and volume was observed (*p* = 0.1657).

These findings suggest that reducing the injectate volume by 50% was not associated with a statistically significant reduction in analgesic duration when dexamethasone was used, suggesting that adjuvant selection may be more important than local anesthetic volume in prolonging postoperative analgesia.

### 3.3. Secondary Analgesic Outcomes

Total 48-h opioid consumption differed significantly according to dexamethasone use but not injectable volume.

In a two-way analysis of variance, perineural dexamethasone was associated with a significant reduction in opioid consumption (mean difference −3.88 mg OME; 95% CI −4.81 to −2.96; *p* < 0.0001), whereas injectate volume (10 mL vs. 20 mL) had no significant effect (*p* = 0.1267). No significant interaction between dexamethasone and injectate volume was observed (*p* = 0.1657).

Descriptive analysis demonstrated lower opioid requirements in dexamethasone-containing groups compared with non-dexamethasone groups, while no statistically significant difference was observed between the 10 mL and 20 mL protocols.

Two-way ANOVA demonstrated a consistent and significant effect of perineural dexamethasone on postoperative pain scores at all time points (4, 8, 12, 24, and 48 h; all *p* < 0.0001). In contrast, injectate volume (10 mL vs. 20 mL) had no significant effect on pain scores at any time point (all *p* > 0.33). No significant interaction between dexamethasone use and injectate volume was observed (all *p* > 0.75), indicating that the analgesic benefit of dexamethasone was independent of local anesthetic volume (Table 3).

### 3.4. Motor Function and Safety Outcomes

Quadriceps muscle strength was preserved in all groups. No clinically relevant motor impairment was detected (Table 4). No nerve injury was identified in any patient at 12, 24, or 48 h postoperatively. All nerve damage scores remained at N0 throughout the observation period, indicating the absence of sensory or motor neurological complications.

### 3.5. Inflammatory and Metabolic Markers

Markers of systemic inflammation showed modest but consistent trends favoring dexamethasone-containing protocols. NLR values at 12, 24, and 48 h were lower in the PENG + DEX groups than in the non-dexamethasone groups, with statistically significant differences observed, particularly between the low-volume PENG 10 mL and PENG 10 mL + DEX groups at 12 and 24 h. PLR values did not differ significantly between groups at any time point.

Blood glucose levels remained within a clinically acceptable range in all groups. Although small between-group differences were noted at selected time points, no consistent or clinically relevant hyperglycemic effect associated with perineural dexamethasone was observed (Table 5).

## 4. Discussion

The present randomized, quadruple-blinded clinical trial demonstrates that the addition of perineural dexamethasone to the pericapsular nerve group (PENG) block significantly prolongs postoperative analgesia and reduces opioid consumption following total hip arthroplasty (THA), regardless of the volume of local anesthetic used. The most important finding of this study is that a low-volume PENG block (10 mL of 0.2% ropivacaine) combined with dexamethasone did not demonstrate a statistically significant difference in analgesic efficacy between the low- and standard-volume approaches. Importantly, the absence of a statistically significant difference should not be interpreted as evidence of equivalence or non-inferiority.

PENG blocks supplemented with dexamethasone resulted in a clinically and statistically significant prolongation of time to first rescue opioid administration compared with ropivacaine alone. This finding is consistent with growing evidence supporting the analgesic-enhancing properties of perineural dexamethasone in peripheral nerve blocks [8,11,12]. Importantly, the absence of a significant difference between the PENG 10 mL + DEX and PENG 20 mL + DEX groups indicates that the analgesic benefit was driven primarily by the adjuvant rather than by local anesthetic volume [14]. The magnitude of the observed effect (HR 0.08) appears large compared with previous literature on dexamethasone as an adjuvant [8,12,15]. However, this may be explained by the high proportion of censored observations in the dexamethasone groups, where many patients remained opioid-free at 24 h. The use of penalized Cox regression allowed stable estimation under quasi-complete separation, but may also contribute to relatively narrow confidence intervals. Therefore, these estimates should be interpreted cautiously.

This observation is particularly relevant in the context of fascial plane blocks, where higher injectate volumes are often empirically used to ensure adequate spread [16]. Recent anatomical and imaging studies suggest that the PENG block targets articular branches of the femoral, obturator, and accessory obturator nerves within a confined pericapsular space, potentially allowing for effective analgesia with smaller volumes when block placement is precise [17]. Our findings support the concept that dexamethasone may compensate for reduced volume by prolonging neural blockade and modulating local inflammatory responses [14]. Compared with the fascia iliaca compartment block (FICB), which has been shown to provide effective analgesia after total hip arthroplasty, the PENG block may offer superior motor preservation; however, direct comparative data remain limited, and further studies are needed to clarify these differences [18].

Total opioid consumption during the first 48 postoperative hours was significantly reduced in the dexamethasone-containing groups, with no statistically significant difference between low- and standard-volume PENG protocols. Given the well-documented risks associated with perioperative opioid use in older patients undergoing THA—including delirium, respiratory depression, nausea, and delayed mobilization—this opioid-sparing effect is clinically important [19,20,21]. Although total opioid consumption is commonly used as a surrogate marker of analgesic efficacy, it does not necessarily reflect patient satisfaction [19,20]. Postoperative nausea and vomiting (PONV), which may be dose-dependent or related to individual patient predisposition, can significantly influence patient experience and may be more distressing than pain itself. This aspect was not assessed in the present study and warrants further investigation [22,23].

Notably, pain scores were consistently lower in the dexamethasone groups across all time points up to 48 h, reinforcing the durability of the analgesic benefit [24]. The similarity of pain trajectories between the two dexamethasone groups further strengthens the argument for volume reduction without loss of efficacy.

Preservation of quadriceps strength is a cornerstone of modern analgesic strategies for hip arthroplasty, as early mobilization is directly linked to improved functional recovery and reduced complication rates [2]. In this study, quadriceps strength was fully preserved in all groups from 12 h postoperatively onward, with only minimal and transient differences at early time points. Crucially, the use of dexamethasone did not increase the risk of motor weakness, even when combined with a higher injectate volume.

No nerve injury or neurological complications were observed in any study group, supporting the safety of both volume strategies and the perineural use of dexamethasone in this setting [14,25]. These findings align with recent systematic reviews indicating a favorable safety profile for perineural dexamethasone at clinically appropriate doses [12].

Markers of systemic inflammation, particularly the neutrophil-to-lymphocyte ratio (NLR), were modestly lower in dexamethasone-containing groups, with statistically significant differences observed in the low-volume comparison. Although the magnitude of these changes was small, they suggest a potential anti-inflammatory effect of perineural dexamethasone extending beyond analgesia [26,27]. This observation is biologically plausible, as dexamethasone is known to reduce local cytokine release and neural inflammation, which may, in turn, indirectly influence systemic inflammatory markers [28]. No adjustment for multiple comparisons was applied to exploratory analyses of inflammatory markers, which increases the risk of type I error and false-positive findings. The observed differences in inflammatory markers, particularly NLR, should be interpreted as exploratory findings, as the study was not powered to detect them, and their clinical relevance remains uncertain.

Blood glucose levels remained stable and within clinically acceptable ranges in all groups, and no consistent hyperglycemic effect attributable to perineural dexamethasone was observed. This is an important safety consideration, particularly in an elderly population with a high prevalence of impaired glucose tolerance [29].

Taken together, these findings support a paradigm shift toward volume optimization rather than escalation in PENG block protocols. The combination of low-volume ropivacaine with perineural dexamethasone achieves effective and prolonged analgesia while potentially reducing the risks associated with higher local anesthetic volumes, such as unintended spread, systemic toxicity, or motor blockade [5,16,30,31].

From a practical standpoint, this strategy aligns well with enhanced recovery after surgery (ERAS) pathways and opioid-sparing anesthesia concepts. It may be particularly advantageous in frail or elderly patients, in whom minimizing drug exposure without compromising analgesia is a key objective [32,33]. The precise mechanism underlying the observed effect remains uncertain. While dexamethasone is known to prolong block duration through anti-inflammatory and neural modulatory pathways, the relative contribution of pharmacological effects versus potential differences in injectate spread within the pericapsular space cannot be determined from the present study.

Importantly, this study was not designed as a non-inferiority or equivalence trial, and no predefined non-inferiority margin was specified. Therefore, the absence of statistically significant differences between volume groups should be interpreted cautiously and considered hypothesis-generating rather than confirmatory.

Several limitations should be acknowledged. This was a single-center study, which may limit generalizability. Although the sample size was sufficient for the primary outcome, the study was not powered to detect rare adverse events. Exclusion of patients with chronic opioid use may limit generalizability, as this population represents a clinically relevant subset of patients undergoing THA. In addition, inflammatory markers were limited to routinely available indices and may not fully capture the complexity of the perioperative immune response. Also, the single-center design and the standardized postoperative analgesic regimen, including the absence of NSAIDs, may limit the generalizability of these findings to institutions using different multimodal analgesia strategies.

## 5. Conclusions

Perineural dexamethasone significantly improves the analgesic efficacy of the PENG block after total hip arthroplasty. A reduced-volume approach did not demonstrate a statistically significant difference in analgesic outcomes compared with standard-volume protocols; however, these findings should be interpreted cautiously, as the study was not designed to assess non-inferiority.

## Figures and Tables

**Table 1:** Baseline characteristics of the study participants.

Variable	PENG 20 mL (n = 30)	PENG 20 mL + DEX (n = 30)	PENG 10 mL (n = 30)	PENG 10 mL + DEX (n = 30)	*p*-Value
Age (years), mean ± SD	67.5 ± 7.1	66.9 ± 6.8	68.2 ± 7.4	67.1 ± 6.9	0.721
Sex (Male/Female), n (%)	16/14 (53/47)	15/15 (50/50)	17/13 (57/43)	14/16 (47/53)	0.876
BMI (kg/m^2^), mean ± SD	27.4 ± 3.2	27.1 ± 3.5	27.6 ± 3.0	27.2 ± 3.3	0.833
ASA Physical Status (II/III), n	18/12	19/11	17/13	20/10	0.794
Duration of surgery (min), mean ± SD	92.1 ± 15.3	93.4 ± 16.0	91.7 ± 14.8	94.0 ± 15.7	0.912
Side of surgery (Right/Left), n	15/15	16/14	15/15	14/16	0.938

Data are presented as mean ± standard deviation (SD) or number (percentage), as appropriate. BMI—body mass index; ASA—American Society of Anesthesiologists physical status classification; n—number of patients. *p*-value compares all four groups. Continuous variables were analyzed using one-way ANOVA. Categorical variables were analyzed using the chi-square test or Fisher’s exact test, as appropriate. Significant *p*-value (*p* < 0.05).

**Table 2:** Analgesic outcomes.

Outcome	PENG 20 mL	PENG 20 mL + DEX	PENG 10 mL	PENG 10 mL + DEX	DEX Effect (*p*-Value)	Volume Effect (*p*-Value)	Interaction (*p*-Value)
Time to first rescue opioid (h)	9.1 ± 1.7	15.0 ± 1.5	8.8 ± 1.6	14.6 ± 1.4	NA	NA	NA
Total 48 h opioid consumption (mg OME)	5.0 ± 4.4	2.3 ± 3.1	6.2 ± 3.8	2.7 ± 2.9	**<0.0001**	0.1267	0.1657

h—hours; OME—oral morphine equivalent; NA—not applicable. Data for time to first rescue opioid are shown for descriptive purposes only; Inferential analysis of the primary outcome was based on Kaplan–Meier estimates, log-rank testing, and a penalized Cox proportional hazards model. *p*-values for the primary outcome were derived from the penalized Cox regression model and are reported in the main text; therefore, they are not presented in this table.

**Table 3:** Pain score outcomes.

Outcome	PENG 20 mL	PENG 20 mL + DEX	PENG 10 mL	PENG 10 mL + DEX	DEX Effect (*p*-Value)	Volume Effect (*p*-Value)	Interaction (*p*-Value)
NRS 4 h	3.2 ± 1.0	2.2 ± 0.8	3.4 ± 0.9	2.3 ± 0.7	**<0.0001**	0.3399	0.7500
NRS 8 h	2.8 ± 0.9	2.0 ± 0.7	2.9 ± 0.8	2.1 ± 0.6	**<0.0001**	0.4716	>0.9999
NRS 12 h	2.5 ± 1.1	1.7 ± 0.8	2.7 ± 1.0	1.8 ± 0.7	**<0.0001**	0.3705	0.7649
NRS 24 h	2.3 ± 0.8	1.5 ± 0.6	2.4 ± 0.9	1.6 ± 0.5	**<0.0001**	0.4469	>0.9999
NRS 48 h	1.9 ± 0.7	1.2 ± 0.5	2.0 ± 0.7	1.3 ± 0.5	**<0.0001**	0.3697	>0.9999

NRS—numerical rating scale (0 = no pain, 10 = worst imaginable pain); h—hours; DEX—dexamethasone. Data are presented as mean ± standard deviation (SD). Pain scores at each time point were analyzed using two-way analysis of variance with dexamethasone use and injectate volume as factors.

**Table 4:** Motor function and nerve damage outcomes.

Outcome	PENG 20 mL	PENG 20 mL + DEX	PENG 10 mL	PENG 10 mL + DEX	DEX Effect (*p*-Value)	Volume Effect (*p*-Value)	Interaction (*p*-Value)
Quadriceps strength 4 h	5.0 ± 0.0	5.0 ± 0.0	5.0 ± 0.0	5.0 ± 0.0	—	—	—
Quadriceps strength 8 h	5.0 ± 0.0	5.0 ± 0.0	5.0 ± 0.2	5.0 ± 0.0	—	—	—
Quadriceps strength 12–48 h	5.0 ± 0.0	5.0 ± 0.0	5.0 ± 0.0	5.0 ± 0.0	—	—	—
Nerve damage 12 h	0.0 ± 0.0	0.0 ± 0.0	0.0 ± 0.0	0.0 ± 0.0	—	—	—
Nerve damage 24 h	0.0 ± 0.0	0.0 ± 0.0	0.0 ± 0.0	0.0 ± 0.0	—	—	—
Nerve damage 48 h	0.0 ± 0.0	0.0 ± 0.0	0.0 ± 0.0	0.0 ± 0.0	—	—	—

h—hours; DEX—dexamethasone; Data are presented as mean ± standard deviation (SD). Quadriceps strength was assessed using a 5-point muscle strength scale (0 = no contraction, 5 = normal strength). Nerve damage was defined as the presence of new sensory or motor deficit in the operated limb. Continuous variables were analyzed using two-way analysis of variance with dexamethasone use and injectate volume as factors. Categorical variables were analyzed using the chi-square test or Fisher’s exact test, as appropriate.

**Table 5:** Inflammatory and metabolic response markers.

Outcome	PENG 20 mL	PENG 20 mL + DEX	PENG 10 mL	PENG 10 mL + DEX	DEX Effect (*p*-Value)	Volume Effect (*p*-Value)	Interaction (*p*-Value)
NLR 12 h	2.0 ± 0.7	1.7 ± 0.5	2.1 ± 0.8	1.6 ± 0.6	**0.0012**	1.0000	0.4080
NLR 24 h	2.0 ± 0.7	1.7 ± 0.5	2.1 ± 0.8	1.6 ± 0.6	**0.0012**	1.0000	0.4080
NLR 48 h	1.8 ± 0.6	1.6 ± 0.5	1.9 ± 0.7	1.5 ± 0.6	**0.0075**	1.0000	0.3665
PLR 12 h	181.4 ± 45.0	187.9 ± 70.9	179.5 ± 40.2	185.0 ± 67.0	0.5677	0.8191	0.9620
PLR 24 h	181.4 ± 45.0	187.9 ± 70.9	179.5 ± 40.2	185.0 ± 67.0	0.5677	0.8191	0.9620
PLR 48 h	180.3 ± 44.1	183.5 ± 65.4	178.6 ± 39.8	180.0 ± 62.7	0.8165	0.7931	0.9276
Blood glucose 12 h	112.3 ± 11.5	113.6 ± 12.1	111.5 ± 10.9	114.2 ± 11.3	0.3410	0.9620	0.7385
Blood glucose 24 h	108.9 ± 10.7	110.1 ± 11.2	107.8 ± 10.5	109.3 ± 10.2	0.4891	0.6263	0.9387
Blood glucose 48 h	104.6 ± 9.8	105.7 ± 10.3	104.2 ± 10.0	105.1 ± 9.7	0.5832	0.7837	0.9562

h—hours; NLR—neutrophil-to-lymphocyte ratio; PLR—platelet-to-lymphocyte ratio; DEX—dexamethasone. Data are presented as mean ± standard deviation (SD). Continuous variables were analyzed using two-way analysis of variance with dexamethasone use and injectate volume as factors.

## Data Availability

The datasets generated and/or analyzed during the current study are available from the corresponding author on reasonable request. The data are not publicly available due to ethical and privacy restrictions concerning patient data.

## References

[1] Patel I., Nham F., Zalikha L., El-Othmani M.M. (2023). Epidemiology of total hip arthroplasty: Demographics, comorbidities and outcomes. Arthroplasty.

[2] Reysner T., Kowalski G., Reysner M., Mularski A., Daroszewski P., Wieczorowska-Tobis K. (2025). Functional recovery and pain control following Pericapsular Nerve Group (PENG) block following hip surgeries: A systematic review and meta-analysis of randomised controlled trials. Arch. Orthop. Trauma Surg..

[3] Pepper C.G., Mikhaeil J.S., Khan J.S. (2024). Perioperative regional anesthesia on persistent opioid use and chronic pain after noncardiac surgery: A systematic review and meta-analysis of randomized controlled trials. Anesth. Analg..

[4] Reysner T., Kowalski G., Reysner M., Lapaj L., Daroszewski P., Wieczorowska-Tobis K. (2025). Erector spinae plane block (ESPB) vs. pericapsular nerve group (PENG) block in total hip arthroplasty in elderly patients: A randomized, double-blinded, controlled trial. Anaesthesiol. Intensive Ther..

[5] Huang Y., Lu Y., Wang J., Lu Q., Bao H.-F., Liu L., Dong C.-S. (2024). Effect of Pericapsular Nerve Group Block with Different Concentrations and Volumes of Ropivacaine on Functional Recovery in Total Hip Arthroplasty: A Randomized, Observer-Masked, Controlled Trial. J. Pain Res..

[6] Girón-Arango L., Peng P. (2025). Pericapsular nerve group (PENG) block: What have we learned in the last 5 years?. Reg. Anesth. Pain Med..

[7] Pascarella G., Costa F., Strumia A., Ruggiero A., Agrò F.E., Carassiti M., Cataldo R. (2025). Pericapsular nerve group (PENG) block: What do we have still to learn for recommending its use in clinical practice?. Reg. Anesth. Pain Med..

[8] Albrecht E., Renard Y., Desai N. (2024). Intravenous versus perineural dexamethasone to prolong analgesia after interscalene brachial plexus block: A systematic review with meta-analysis and trial sequential analysis. Br. J. Anaesth..

[9] De Cassai A., Santonastaso D.P., Coppolino F., D’Errico C., Melegari G., Dost B., Aviani Fulvio G., Boscolo A., Boscolo-Berto R., Navalesi P. (2025). Perineural dexamethasone: Neurotoxicity or neuroprotection? A systematic review of preclinical evidence. J. Anesth. Analg. Crit. Care.

[10] Abdildin Y.G., Tapinova K., Nabidollayeva F., Viderman D. (2023). Epidural dexamethasone for acute postoperative pain management: A systematic review with meta-analysis. Pain Manag..

[11] Bei T., Liu J., Huang Q., Wu J., Zhao J. (2021). Perineural versus intravenous dexamethasone for brachial plexus block: A systematic review and meta-analysis of randomized controlled trials. Pain Physician.

[12] Zufferey P.J., Chaux R., Lachaud P.-A., Capdevila X., Lanoiselée J., Ollier E. (2024). Dose–response relationships of intravenous and perineural dexamethasone as adjuvants to peripheral nerve blocks: A systematic review and model-based network meta-analysis. Br. J. Anaesth..

[13] Garcia-Gomara M., Juan-Palencia A., Alfaro M., Cuadrado-Tejedor M., Garcia-Osta A. (2024). Neuroprotective effects of dexamethasone in a neuromelanin-driven Parkinson’s disease model. J. Neuroimmune Pharmacol..

[14] Reysner T., Neumann-Podczaska A., Pietraszek P., Mularski A., Kowalski G., Daroszewski P., Reysner M. (2025). Reduced Ropivacaine Volume with Perineural Dexamethasone in PENG Block for Total Hip Arthroplasty: A Randomized Controlled Trial. J. Clin. Med..

[15] Dimmen A., Timko S., Greenwood J., McShane F., Ulinski J. (2023). Effect of dexamethasone administration for postoperative nausea and vomiting prophylaxis on glucose levels in adults with diabetes undergoing elective surgery: A systematic review with meta-analysis. JBI Evid. Synth..

[16] Yeoh S.-R., Chou Y., Chan S.-M., Hou J.-D., Lin J.-A. (2022). Pericapsular nerve group block and iliopsoas plane block: A scoping review of quadriceps weakness after two proclaimed motor-sparing hip blocks. Healthcare.

[17] Kim J.Y., Kim J., Kim D.-H., Han D.W., Kim S.H., Kim D., Chung S., Yu S., Lee U.-Y., Park H.J. (2023). Anatomical and radiological assessments of injectate spread stratified by the volume of the pericapsular nerve group block. Anesth. Analg..

[18] Gola W., Bialka S., Owczarek A.J., Misiolek H. (2021). Effectiveness of fascia iliaca compartment block after elective total hip replacement: A prospective, randomized, controlled study. Int. J. Environ. Res. Public Health.

[19] Sun X.-J., Feng T.-C., Wang Y.-M., Wang F., Zhao J.-B., Liu X., Li F.-L. (2023). The effect of the enhanced recovery after surgery protocol and the reduced use of opioids on postoperative outcomes in elderly patients with colorectal cancer. Eur. Rev. Med. Pharmacol. Sci..

[20] He Z., Kangjie K., Huang Z., Fang J. (2022). Effect of reducing-opioids consumption on postoperative delirium incidence in elderly patients after gastric cancer surgery. Zhonghua Yi Xue Za Zhi.

[21] Liu Q., Li L., Wei J., Xie Y. (2023). Correlation and influencing factors of preoperative anxiety, postoperative pain, and delirium in elderly patients undergoing gastrointestinal cancer surgery. BMC Anesthesiol..

[22] Riveros-Perez E., Bolanos J.A., Gomez P., Avella-Molano B., Pulijal V. Dose-Response Relationship of Dexamethasone for Postoperative Nausea and Vomiting: A Retrospective Stratified Analysis Based on Apfel Score. https://assets-eu.researchsquare.com/files/rs-7614826/v1/3669f7d4-027f-4b18-b55f-5bf24f11a287.pdf?c=1773241064.

[23] Öbrink E. (2025). Post Operative Nausea and Vomiting: The Big Little Problem Impact on Recovery.

[24] Viderman D., Tapinova K., Aryngazin A., Aubakirova M., Abdildin Y. (2025). Perineural dexamethasone added to peripheral nerve block in knee surgery: A systematic review with meta-analysis. Anaesthesiol. Intensive Ther..

[25] Reysner T., Kowalski G., Mularski A., Pyszczorska M., Grochowicka M., Perek A., Daroszewski P., Reysner M. (2025). Perineural dexamethasone effectively prolongs anaesthesic block duration in total hip arthroplasty, reduces opioid consumption, and does not compromise motor function, nerve integrity, or glycaemic control. Int. Orthop..

[26] He R., Li X., Zhang S., Liu Y., Xue Q., Luo Y., Yu B., Li X., Liu Z. (2023). Dexamethasone inhibits IL-8 via glycolysis and mitochondria-related pathway to regulate inflammatory pain. BMC Anesthesiol..

[27] Singh N.P., Makkar J.K., Chawla J.K., Sondekoppam R.V., Singh P.M. (2024). Prophylactic dexamethasone for rebound pain after peripheral nerve block in adult surgical patients: Systematic review, meta-analysis, and trial sequential analysis of randomised controlled trials. Br. J. Anaesth..

[28] Couch B., Hayward D., Baum G., Sakthiyendran N.A., Harder J., Hernandez E.J., MacKay B. (2024). A systematic review of steroid use in peripheral nerve pathologies and treatment. Front. Neurol..

[29] Pansini A., Lombardi A., Morgante M., Frullone S., Marro A., Rizzo M., Martinelli G., Boccalone E., De Luca A., Santulli G. (2022). Hyperglycemia and physical impairment in frail hypertensive older adults. Front. Endocrinol..

[30] Macfarlane A., Gitman M., Bornstein K., El-Boghdadly K., Weinberg G. (2021). Updates in our understanding of local anaesthetic systemic toxicity: A narrative review. Anaesthesia.

[31] Maagaard M., Stormholt E.R., Nielsen L.F., Bærentzen F., Danker J., Zachodnik J., Jæger P., Mathiesen O., Andersen J.H. (2023). Perineural and systemic dexamethasone and ulnar nerve block duration: A randomized, blinded, placebo-controlled trial in healthy volunteers. Anesthesiology.

[32] Kianian S., Bansal J., Lee C., Zhang K., Bergese S.D. (2024). Perioperative multimodal analgesia: A review of efficacy and safety of the treatment options. Anesthesiol. Perioper. Sci..

[33] Del Tedesco F., Sessa F., Xhemalaj R., Sollazzi L., Russo C.D., Aceto P. (2023). Perioperative analgesia in the elderly. Saudi J. Anaesth..

